# Ameliorative effect of biosynthesized titanium dioxide nanoparticles using garlic extract on the body weight and developmental toxicity of liver in albino rats compared with chemically synthesized nanoparticles

**DOI:** 10.3389/fvets.2022.1049817

**Published:** 2022-12-16

**Authors:** Zeinab Kamal, A. A. Ebnalwaled, Zeinab Al-Amgad, Alaa H. Said, Asmaa A. Metwally, František Zigo, Silvia Ondrašovičová, Ibrahim F. Rehan

**Affiliations:** ^1^Zoology Department, Faculty of Science, South Valley University, Qena, Egypt; ^2^Electronic and Nano Devise Lab, Faculty of Science, South Valley University, Qena, Egypt; ^3^General Authority for Veterinary Services, Faculty of Veterinary Medicine, South Valley University, Qena, Egypt; ^4^Department of Surgery, Anesthesiology, and Radiology, Faculty of Veterinary Medicine, Aswan University, Aswan, Egypt; ^5^Department of Nutrition and Animal Husbandry, University of Veterinary Medicine and Pharmacy in Košice, Košice, Slovakia; ^6^Department of Biology and Physiology, University of Veterinary Medicine and Pharmacy in Košice, Košice, Slovakia; ^7^Department of Husbandry and Development of Animal Wealth, Faculty of Veterinary Medicine, Menoufia University, Shebin Alkom, Egypt; ^8^Department of Pathobiochemistry, Faculty of Pharmacy, Meijo University, Nagoya-shi, Japan

**Keywords:** Bax-immunohistochemically, garlic, bodyweight, fetuses, histology, liver, TiO_2_NPs

## Abstract

The application of metallic nanoparticles poses risks to human and animal health. Titanium dioxide nanoparticles (TiO_2_NPs) are the most commonly synthesized metallic oxides in the world. Exposure to TiO_2_NPs can cause toxicity in the target organisms. This study aimed to evaluate the effects of green and chemical TiO_2_NPs on maternal and embryo-fetal livers. Green TiO_2_NPs using garlic extract (GTiO_2_NPs) and chemical TiO_2_NPs (CHTiO_2_NPs) were synthesized and characterized by x-ray powder diffraction and high-resolution transmission electron microscopy. The cytotoxicity of both chemical and green TiO_2_NPs was determined against HepG_2_ cell lines. Fifty pregnant female Albino rats were equally and randomly divided into five groups. Group 1 was kept as a control. Groups 2 and 3 were orally treated with 100 and 300 mg/kg body weight of CHTiO_2_NPs, respectively. Groups 4 and 5 were orally treated with 100 and 300 mg/kg of GTiO_2_NPs, respectively, from day 6 to 19 of gestation. All dams were euthanized on gestation day 20. All live fetuses were weighed and euthanized. Blood and tissue samples were collected for biochemical, histopathological, and Bax-immunohistochemical expression analyses. Our results indicated that garlic could be used as a reducing agent for the synthesis of TiO_2_NPs, and the produced NPs have no toxic effect against HepG_2_ cells compared with CHTiO_2_NPs. The maternal and fetal bodyweights were greatly reduced among the chemically TiO_2_NPs induced animals. The mean serum level of AST and ALT activities and the total protein level significantly increased when TiO_2_NPs were administered at high doses. Histologically, the CHTiO_2_NPs-treated groups revealed vacuolated and necrotized hepatocytes with congested and dilated blood vessels in the fetal and maternal livers. The immunohistochemistry revealed distinct positive staining of Bax expressed in the hepatocytes. Nevertheless, the biosynthesis of TiO_2_NPs using garlic extract had a minimal effect on the normal architecture of the liver. It could be concluded that the bioactivity of TiO_2_NPs can be modified by green synthesis using garlic extract. Compared to the CHTiO_2_NPs, the exposure to GTiO_2_NPs showed reduced liver damage in maternal and embryo-fetal rats.

## Graphical Abstract

The schematic cartoon of the experimental design – It was created by BioRender.com. 
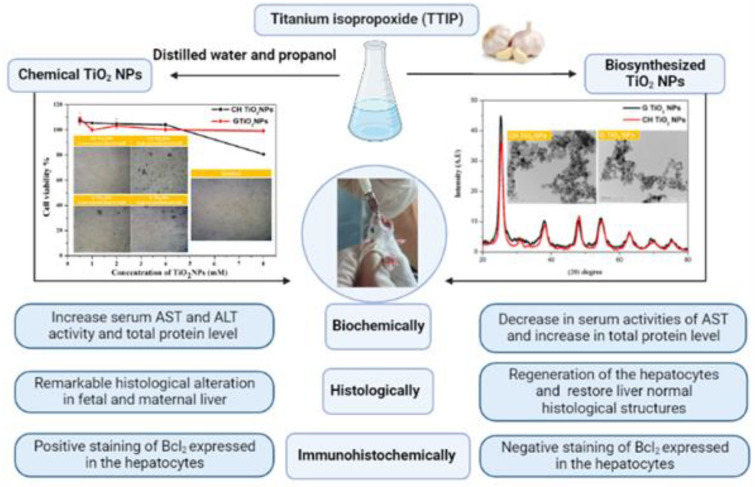

## Introduction

There has been a huge increase in the application of nanoparticles in recent years. The development of nanotechnology offers practical benefits in a variety of industries, including the textile, cosmetics, energy, and chemical sectors (1), when compared with larger-scale particles with the same chemical composition (2). Due to their ease of preparation and low cost of production, titanium dioxide nanoparticles (TiO_2_NPs), among the most well-known metallic oxide NPs, have raised significant concerns (3). Currently, different industrial sectors are using TiO_2_NPs in a number of scientific fields, including the production of plastics, the use of adjuvants in the formulation of pharmaceutical pills, bleaching agents in the paper industry, and the manufacture of paints, cosmetics, sunscreens, and toothpaste (4). The same study claimed that the usage of nanoparticles as food additives is widespread (5). Ingestion, inhalation, injection into the skin, and penetration were the most common routes of exposure (6). Despite their widespread use and daily exposure, the effects of these particles on animal and human health and the environment remain unknown. Lately, The public health dangers associated with exposure to silica or metallic oxide nanoparticles were the first to suggest the adverse biological response of nano-sized materials (7, 8). We could argue that nanotechnology has become a double-edged sword.

While it has the potential to enhance people's quality of life, it also has some downsides: as environmental contamination increases, it becomes hazardous and poses a serious threat to living organisms. Chemically, NPs are more reactive than heavy metals because of their own physicochemical characteristics (9). In toxicological profiles due to metallic nanoparticle (MNP) exposure, the deteriorations in the levels of the specific enzyme directly reflect the damage in specific organs (6, 10). TiO_2_NPs were determined to induce hepatocyte apoptosis and increase the inflammatory response (11). An increase in the expression of the genes for reactive oxygen species (ROS) and cytochrome p450 is thought to be the cause of liver damage (CYP1A). Following this process, activated hepatic macrophages produce inflammatory mediators, resulting in lipid peroxidation and protein oxidation, which cause hepatocellular necrosis (12).

Green synthetic nanoparticles have been offered as new avenues for nanotechnology advancement because they are safer and more environmentally friendly. Biological agents such as bacteria, plant extracts, and fungi are increasingly being used in green synthesis instead of serious chemical agents (13, 14) and algae (15). Cost-effectiveness and a one-step process are advantages of green synthesis (16). Various extracts from plant origins have previously been used in green synthetic nanoparticles due to their easy preparation (17). Scientific reviewers suggest that plant-based extracts may naturally possess hepatoprotective properties. Garlic (*Allium sativum*) can provide a histo-protective role against organ damage caused by NPs, particularly liver damage (18). Some constituents isolated from garlic, such as steroids, terpenoids, polyphenols, flavonoids, and other phenolic chemicals, have been demonstrated to shield the liver against harmful substances (19).

As a consequence, a diet high in flavonoids can lower the risk of tissue degradation and change caused by specific disorders (20). The possibility of fetal malformations and deaths is dramatically increased by exposure to TiO_2_NPs. However, research conducted on pregnant mice to examine the possibility of TiO_2_NPs toxicity on placental growth and development is scant (21). Therefore, this study is a trial to spot the harmful effect of CHTiO_2_NPs on the liver of the maternal rats and their embryos and how GTiO_2_NPs could alleviate hepatotoxicity.

## Materials and methods

### Chemical synthesis of TiO_2_NPs

According to the previous report (22), the preparation of CHTiO_2_NPs was performed using the chemical coprecipitation method. Titanium isopropoxide (TTIP) was purchased from Sigma Co., USA; it has been used as a precursor for TiO_2_, while a mixture of distilled water and propanol was used as a solvent with a 50/1 (V/V). To obtain the solvent solution, 250 ml of water and 5 ml of propanol were combined, and to produce the precursor solution 15 ml of propanol and 5 ml of TTIP were combined. After being heated at 70°-90°C under constant stirring for 2 h, the solvent solution was modified by adding the precursor solution drop by drop. A white precipitate was observed immediately, which indicated the reduction of TiO_2_. After leaving it to cool overnight at 25°C, this precipitate was washed three times with distilled water and one time with ethanol. The prepared sample was washed, dried for 12 h at 100°C, and then calcinated for 3 h at 400°C.

### Green synthesis of TiO_2_NPs

Garlic cloves were purchased from the local market belonging to Qena City, Qena, Egypt. To make GTiO_2_NPs, 20 g of dried, finely ground garlic was boiled in 150 ml of distilled water for 1 h. The TiO_2_ precursor solution was made by vigorously stirring 150 ml of distilled water with 10 ml of TTIP. Then, 60 ml of fresh garlic plant extract was addedto the TiO_2_ precursor solution with continuous stirring for 2 h. The solution's color changed from white to dark yellow, which signaled the mitigation of TTIP and the formation of GTiO_2_NPs. The created NPs were dried at 100°C and cooled at 25°C overnight. Finally, the sample was finely crushed into powder using an agate mortar and pestle and then calcinated at 450°C for 2 h (23).

### TiO_2_NPs characterization

The prepared TiO_2_NPs were characterized by x-ray diffraction (XRD) following X'Pert PRO-PAN analytical diffractometer with Cu-Kα radiation (λ = 1.54056A°), which was produced at 40 kV and 30 mA. Using high-resolution transmission electron microscopy (HRTEM), the samples' morphology was discovered (JEOL, JEM 2100, Japan).

### Cytotoxicity of TiO_2_NPs

Liver hepatocellular cells (HepG_2_ cell lines) were employed to assess the toxicity of TiO_2_NPs following an MTT assay. HepG_2_ cell lines were purchased from Vacsera (Giza, Egypt). In DMEM with 10% heat-inactivated fetal bovine serum and 1% penicillin-streptomycin, HepG_2_ cells were grown.

A total of 1 × 10^4^ HepG_2_ cells were seeded into 96-well culture plates, incubating at 37°C and 5% CO_2_. By dissolving “3-(4,5-Dimethyl-2-thiazolyl)-2,5-diphenyl-2H-tetrazolium bromide” in PBS at a concentration of 5 mg/mL and mixing the solution while stirring for 1 h, the MTT 3-(4,5-dimethylthiazol-2-yl)-2, 5-diphenyltetrazolium reagent was created. The solution was then filtered, serialized, and stored in the dark at 4°C. The MTT assay was performed according to the standardized protocol (11). The developed cells were briefly rinsed with PBS, exposed to TiO_2_NPs at various concentrations (0, 0.5, 1, 2, 4, and 8 mM), and then incubated for 24 h at 37°C and 5% CO_2_. The exposed cells were rinsed in PBS the following day, subjected to an MTT solution (80 mL of serum-free medium and 20 mL of MTT), and then incubated for 3 h at 37°C and 5% CO_2_. We poured MTT solvent (DMSO) into each well to stop the reaction. Plates were then wrapped in foil and shaken on an orbital shaker for 15 min. Finally, the absorbance was recorded at OD = 590 nm using a Tecan infinite F50 absorbance microplate reader. The experiment was carried out three times, and the outcomes are the three replicates' averages with standard deviations normalized to the control. The percent of cell viability was calculated using the following equation (11):


$$\begin{array}{ll} Cell\;viability\%\;=\; & \frac{Absorbance\;control-Absorbance\;sample}{Absorbance\;control} \end{array}$$

### Experimental animals and management

Fifty adult female Sprague–Dawley Albino rats of 5–6 months' age and 180–220 g average body weight were purchased from the animal house of the National Research Center Institute (Cairo, Egypt). The rats were housed in clean, well-ventilated cages under appropriate temperature and humidity laboratory conditions. Throughout the experiment, all rats had access to conventional food and unlimited access to water. Before the experiment, they had an acclimation period of 2 weeks.

### Experimental design and treatment

For developmental toxicity studies, every three female rats mated with one male rat in a stainless-steel cage at night. The vaginal smear determined the first day of gestation. On day 6 of gestation, pregnant female rats were immediately introduced into the experimental design.

In this experiment, 50 pregnant female Albino rats were randomly assigned to five groups (*n* = 10 each):

- Group 1 was utilized as control and supplied with distilled water.
- Group 2 (CH100) orally received 100 mg/kg bwt of CHTiO_2_NPs.
- Group 3 (CH300) orally received 300 mg/kg bwt of CHTiO_2_NPs.
- Group 4 (G100) orally received 100 mg/kg bwt of GTiO_2_NPs.
- Group 5 (G300) orally received 300 mg/kg bwt of GTiO_2_NPs.

All animals were orally administered fresh suspensions of TiO_2_NPs by intragastric gavage daily from 6 to 19 days of gestation.

During the experiments, all pregnant females were carefully inspected daily during the gestation period for any clinical signs or abnormalities. Moreover, the maternal body weight was measured on days 6, 9, 12, 15, 17, and 20 of gestation. The body weight of fetuses was also recorded.

### Blood samples

On day 20 of gestation, all female rats were euthanized after they were made to inhale a dose of diethyl ether. Using the retro-orbital technique, whole blood samples were drawn, and the serum was obtained in clean, dry tubes, centrifuged at 3,000 rpm for 10 min, and then frozen at −20°C until further biochemical analysis.

### Liver biopsy

The livers of mothers and fetuses were excised and preserved in 10% buffered neutral formalin for histopathological and immunohistochemical studies.

### Biochemical analysis

#### Estimation of serum transaminases (IU/l)

Serum transaminase activities (AST and ALT) were estimated with biodiagnostic enzymatic kits purchased from Biodiagnostic (Dokki, Giza, Egypt) using the colorimetric method, as described previously by Sanghavin and Jivani (24).

#### Estimation of protein profile (g/dl)

Total protein and albumin test kits from Biodiagnostic (Dokki, Giza, Egypt) were used to measure the serum total protein and albumin based on the protocols that have been outlined (25, 26).

#### Histopathological examination

Systemic autopsies from the livers of each group were dissected and thoroughly immersed in 10% buffered neutral formalin (pH 7.2) for 48 h. Paraffin sections of 4 μm in thickness were stained with hematoxylin and eosin (H&E) stain and inspected under the light microscope (27).

#### Immunohistochemical reaction

According to the previous report, the cryostat sections of the liver from all groups were prepared at 4 μm thickness, deparaffinized, and then stained with an immunohistochemical reaction for Bax (28).

### Statistical analysis

The results were statistically expressed as mean ± standard deviation (SD) by a one-way ANOVA, followed by a *post-hoc* Tukey's test for multiple comparisons between different groups. The level of significance was set at a *p*-value of < 0.05 (29).

## Results

### Characterization of TiO_2_NPs

**Figure 1:**
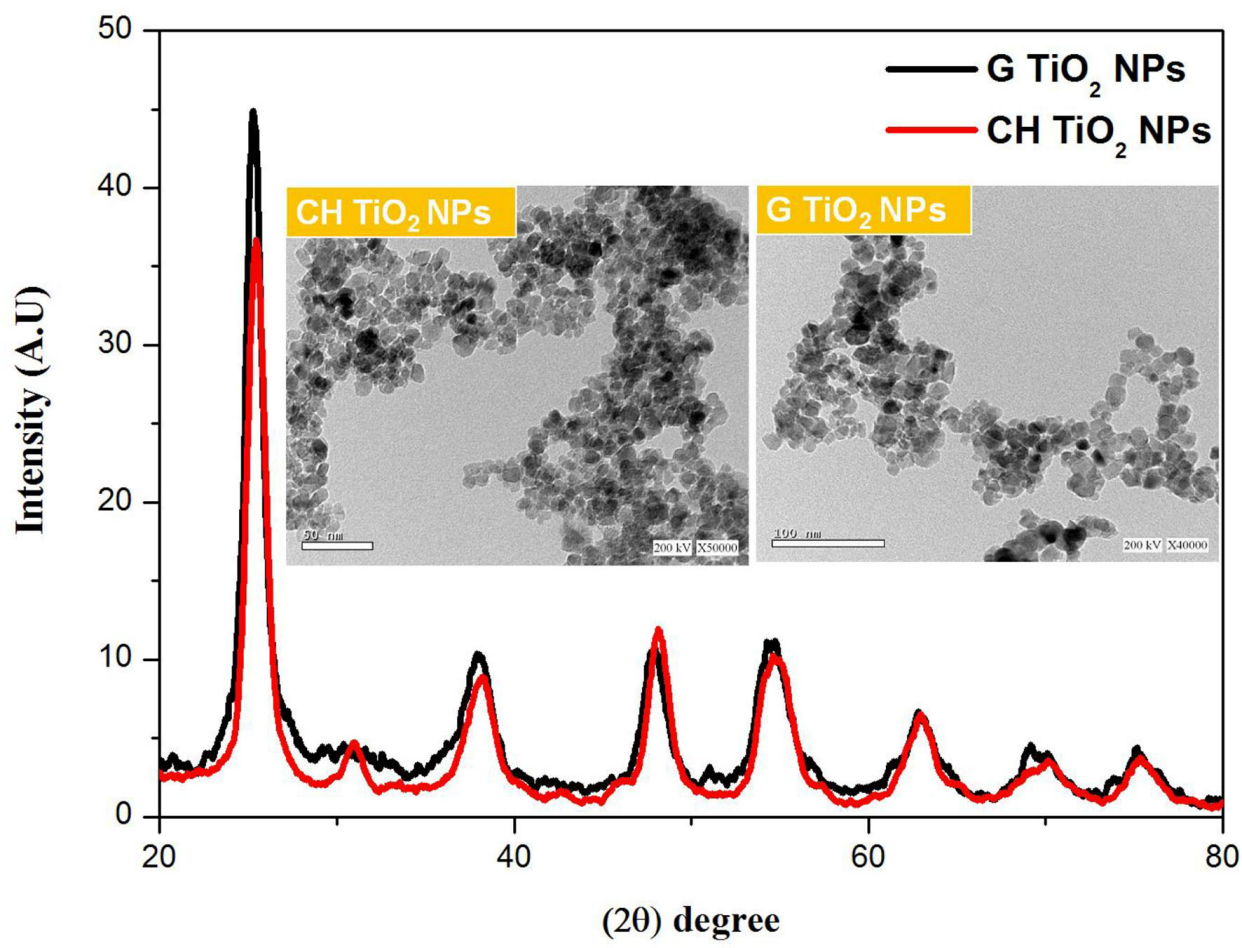
XRD pattern combined with HRTEM images of CHTiO_2_NPs and GTiO_2_NPs.

The -ray diffraction (XRD) pattern of chemically made and environmentally friendly TiO_2_NPs is shown in Figure 1. All the characteristic peaks of TiO_2_NPs were present in the XRD of the two samples, which indicated the successful formation of TiO_2_NPs. The diffraction peaks at 2θ (25.27, 30.9, 37.79, 48, 55.6, 63.3, 70.5, and 74.2) corresponded to the plane (101, 004, 200, 105, 204, and 220, respectively). HRTEM images of TiO_2_NPs showed a sort of agglomeration with a spherical or irregular spherical shape in both samples.

### Cytotoxicity of TiO_2_NPs

**Figure 2:**
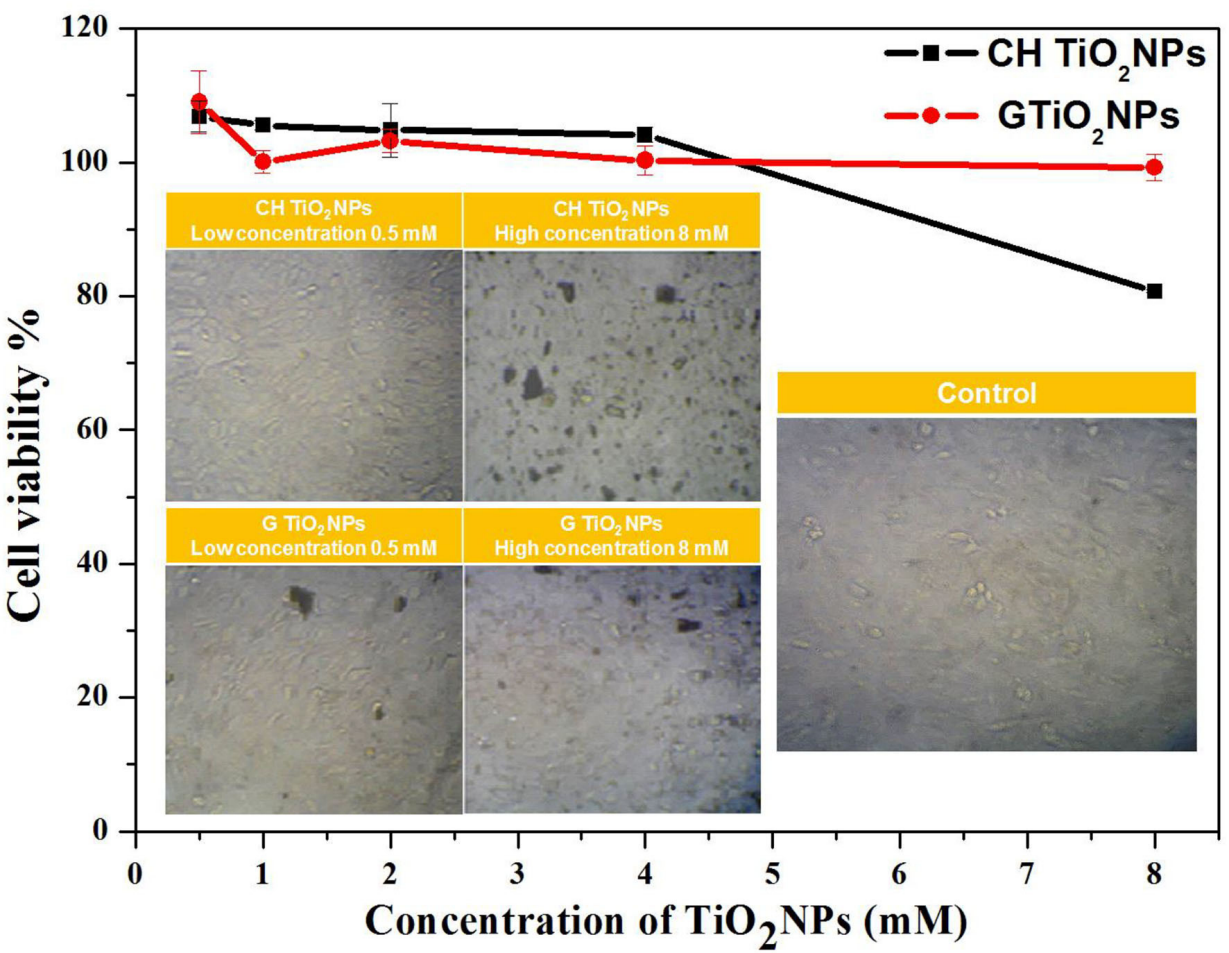
Cell viability of HepG_2_ cells after 24 h exposure to CHTiO_2_NPs and GTiO_2_NPs with different concentrations and microscopic images of HepG_2_ cells after 24 hrs of exposure to CHTiO_2_ NPs and GTiO_2_NPs at concentration 0.5 mM and 8 mM.

The toxicity of TiO_2_NPs against HepG_2_ cells was determined using the MTT assay. Both samples did not exhibit toxicity at low concentrations (0.5–4 mM). However, at a high concentration (8 mM), CHTiO_2_NPs showed a decrease in cell viability (from 104 to 80 %). Moreover, an accumulation of TiO_2_NPs on the surface of the cells was observed at a high concentration, which suggested that the number of NPs internalized in the cells was smaller than the number of adherent NPs, as presented in Figure 2.

### Body weight

#### Maternal body weight

CHTiO_2_NPs 100 and CHTiO_2_NPs 300 significantly decreased maternal body weight from 6 to 20 days of gestation compared to the control group, while GTiO_2_NPs 100 significantly decreased maternal body weight from 6 to 17 days of gestation compared to the control group. Moreover, GTiO_2_NPs 300 considerably decreased at 9, 17, and 20 days of gestation compared to the control group. Additionally, CHTiO_2_NPs 300 significantly increased at 6, 9, 17, and 20 of gestation compared with CHTiO_2_NPs 100. At 6–20 days of gestation, the maternal body weight was considerably higher in GTiO_2_NPs 100 and GTiO_2_NPs 300 than in CHTiO_2_NPs 100, and at 6–15 days of gestation, it was significantly higher in GTiO_2_NPs 100 and GTiO_2_NPs 300 than in CHTiO_2_NPs 300. Furthermore, the maternal body weight of the GTiO_2_NPs 100 treated group significantly increased compared with CHTiO_2_NPs 300 on the 20th day of gestation, while GTiO_2_NPs 300 significantly increased on the 17th day compared with CHTiO_2_NPs 300. Moreover, compared to GTiO_2_NPs 100, GTiO_2_NPs 300 showed a considerable increase between days 6 and 20 of gestation (Table 1).

**Table 1:** Effect of chemical and green TiO_2_NPs on maternal body weight (g/kg) of control and experimental groups.

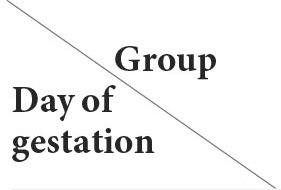	**Control**	**CH 100**	**CH 300**	**G 100**	**G 300**
Day-6	2.26 ± 3.2	1.8 ± 3.0^a^	2 ± 4^a^^,^^b^	2.13 ± 1.0^a^^,^^b^^,^^c^	2.32 ± 4.0^b^^,^^c^^,^^d^
Day-9	2.4 ± 3.5	1.89 ± 2.6^a^	2.11 ± 2.0^a^^,^^b^	2.23 ± 2.0^b^^,^^c^	2.33 ± 1.0^a^^,^^b^^,^^c^^,^^d^
Day-12	2.4 ± 4.4	2.05 ± 2.5^a^	2.19 ± 2.6^a^^,^^b^	2.38 ± 2.0^b^^,^^c^	2.46 ± 2.0^a^^,^^b^^,^^c^^,^^d^
Day-15	2.62 ± 4.5	2.3 ± 2.6^a^	2.32 ± 4.0^a^	2.44 ± 3.6^a^^,^^b^^,^^c^	2.60 ± 2.0^b^^,^^c^^,^^d^
Day-17	2.8 ± 1.0	2.4 ± 4.3^a^	2.5 ± 4.0^a^^,^^b^	2.5 ± 4.0^a^^,^^b^	2.69 ± 1.0^a^^,^^b^^,^^c^^,^^d^
Day-20	2.72 ± 1.0	2.49 ± 1.7^a^	2.6 ± 2.0^a^^,^^b^	2.70 ± 2.0^b^^,^^c^	2.56 ± 2.0^a^^,^^b^^,^^d^

a *P*-value < 0.05 compared with Control.

b *P*-value < 0.05 compared with CH 100.

c *P*-value < 0.05 compared with CH 300.

d *P*-value < 0.05 compared with G 100. CH 100, Chemical TiO_2_NPs of 100 mg/kg; CH 300, Chemical TiO_2_NPs of 300 mg/kg; G 100, greenTiO_2_NPs of 100 mg/kg; G 300, greenTiO_2_NPs of 300 mg/kg.

#### Fetal body weight

Compared with the control, fetal body weight significantly decreased with 300 mg/kg of CHTiO_2_NP, 100 mg/kg of CHTiO_2_NP, 100 mg/kg of GTiO_2_NP, and 300 mg/kg of GTiO_2_NP, as shown in Table 2. Additionally, the number of fetuses significantly decreased CHTiO_2_NP by 300 mg/kg compared with the control.

**Table 2:** Effect of chemical and green TiO_2_NPs on fetal body weight (g/kg) and the number of fetuses of control and experimental groups (mean ± SD).

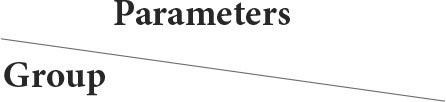	**Fetal body**	**No. of fetuses**
	**Weight**	
Control	5.26 ± 0.6	8.8 ± 0.26
CH 100	4.37 ± 0.3	8.5 ± 0.3
CH 300	2.37 ± 0.6^a^^,^^b^	7.75 ± 0.25^a^
G 100	4.6 ± 0.7^c^	8.25 ± 0.36
G 300	5.3 ± 0.4^c^	8.5 ± 0.36

a *P*-value < 0.05 compared with Control.

b *P*-value < 0.05 compared with CH 100.

c *P*-value < 0.05 compared with CH 300. CH 100, Chemical TiO_2_NPs of 100 mg/kg; CH 300, Chemical TiO_2_NPs of 300 mg/kg; G 100, greenTiO_2_NPs of 100 mg/kg; G 300, greenTiO_2_NPs of 300 mg/kg.

### Biochemical findings

The group treated with 300 mg/kg of CHTiO_2_NPs showed significantly (*P* < 0.05) increased serum AST and ALT activities compared with the control group (Table 3). In contrast, the groups treated with 100 and 300 mg/kg of GTiO_2_NPs showed a significant decrease (*P* < 0.05) in serum AST activity compared with the control group.

**Table 3:** Effect of chemical and green TiO_2_NPs on liver transaminases of control and experimental groups (mean ± SD).

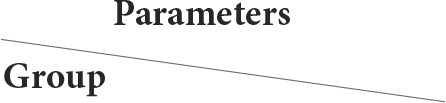	**AST**	**ALT**
Control	119.0 ± 1.4	18.0 ± 1.14
CH 100	93.0 ± 1. 4	17.0 ± 1.0
CH 300	143.0 ± 1.14^a^	34.0 ± 1.14^a^
G 100	74.0 ± 1.1^a^	21.0 ± 1.1
G 300	101.0 ± 1.14^a^	18.0 ± 1.14

a *P*-value < 0.05 compared with Control. CH 100, Chemical TiO_2_NPs of 100 mg/kg; CH 300, Chemical TiO_2_NPs of 300 mg/kg; G 100, greenTiO_2_NPs of 100 mg/kg; G 300, greenTiO_2_NPs of 300 mg/kg.

Regarding the protein profile, total protein exhibited a significant increase (*P* < 0.05) in all exposed groups among the chemical and green TiO_2_NPs compared with the control group. Albumin levels significantly (*P* < 0.05) outperformed the mean values of the treated group with 300 mg/kg of CHTiO_2_NPs in comparison with the control group (Table 4).

**Table 4:** Effect of chemical and green TiO_2_NPs on protein profile of control and experimental groups (mean ± SD).

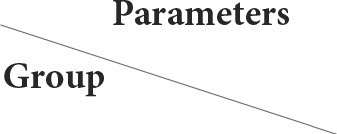	**Total protein** **(g/dl)**	**Albumin** **(g/dl)**
Control	2.9 ± 0.14	3.02 ± 0.14
CH 100	5.8 ± 1.4^a^	3.2 ± 0.14
CH 300	5.5 ± 1. 4^a^	4.1 ± 0.14^a^
G 100	5.0 ± 0.14^a^	2.85 ± 0.14
G 300	6.0 ± 0.14^a^	3.08 ± 0.14

a *P*-value < 0.05 compared with Control. CH100, Chemical TiO_2_NPs of 100 mg/kg; CH 300, Chemical TiO_2_NPs of 300 mg/kg; G 100, greenTiO_2_NPs of 100 mg/kg; G 300, greenTiO_2_NPs of 300 mg/kg.

### Histopathological findings

#### Liver of rat fetuses

**Figure 3:**
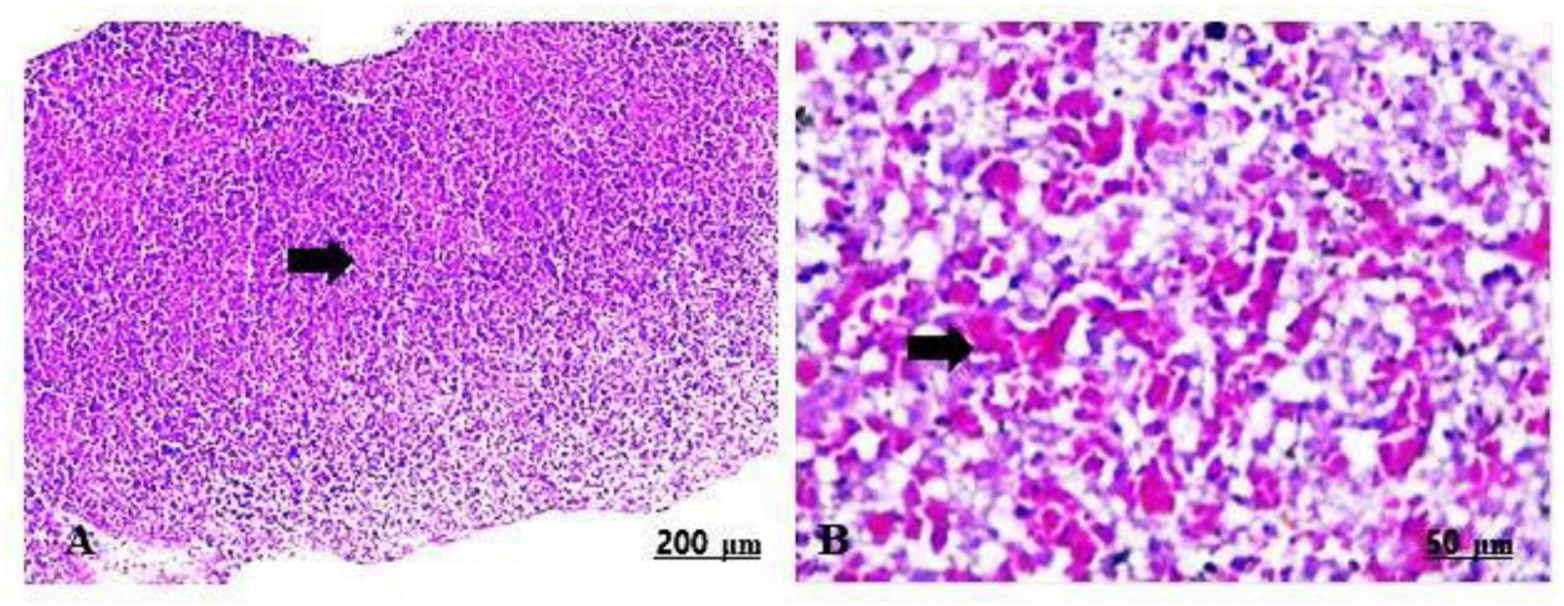
**(A,B)** Light photomicrograph of sections from the fetal liver of control stained with H&E: control showing intense infiltration of hematopoietic cells, mainly RBCs and lymphocytes in the blood sinusoids **(A,B)**.

The fetal livers in the control group showed primitive hepatocytes with prominent infiltration of hematopoietic cells, mostly composed of red blood cells (RBCs) and lymphocytes in the blood sinusoids (Table 5, Figures 3A,B).

**Table 5:** Histopathological and immunostaining scores from the liver of the embryo and mother of control and other experimental groups.

**Groups**	**Control**	**CH 100**	**CH 300**	**G 100**	**G 300**
**Lesions**					
**H&E. stain**					
**Liver of embryo**					
Hepatic necrosis	-	+	++	-	-
Cytoplasmic vacuolization of the hepatocytes	+	++	+++	++	+
Inflammatory cell infiltration	–	+++	+++	+	+
Congestion and dilatation of blood vessels	+	+++	+++	+	+
Proliferation and mitotic division of hepatocytes and blood vessels	++	–	–	++	++
**Liver of adult female (mother)**					
Hepatocytes necrosis	–	+	++	–	–
Hepatic vacuolation	+	++	+++	++	+
Congestion and dilatation of blood vessels	+	+++	+++	+	+
Mononuclear cells infiltration	+	+++	+++	+	+
Regeneration of some hepatocytes	++	+	–	+++	+++
**BCl**_**2**_ **-immunostaining**					
BCl_2_ immunoreactivity in hepatocytes	–	++	+++	–	+

The result represented absent (–), mild (+), moderate (++), and severe (+++). CH100, Chemical TiO_2_NPs of 100 mg/kg; CH300, Chemical TiO_2_NPs of 300 mg/kg; G100, green TiO_2_NPs of 100 mg/kg; G300, green TiO_2_NPs of 300 mg/kg.

**Figure 4:**
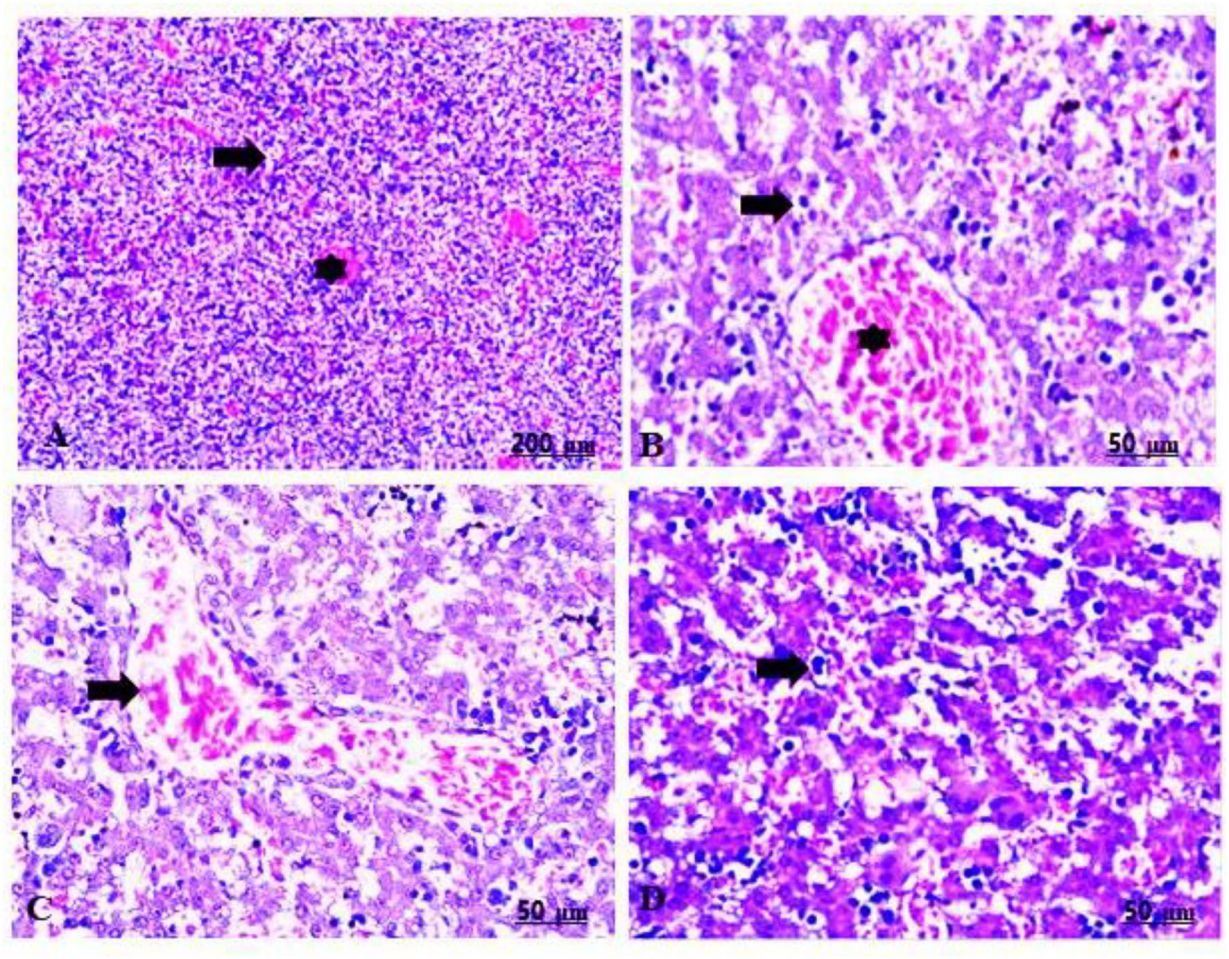
**(A–D)** Light photomicrograph of sections from the fetal liver of CHTiO_2_NPs exposed groups. CHTiO_2_NPs of 100 mg/kg bwt showing extensive hepatic vacuolation (arrow), thrombotic vasculitis of the central vein, and lymphocyte infiltration (star) **(A,B)**. CHTiO_2_NPs of 300 mg/kg bwt show remarkable congestion and dilatation of the blood vessels **(C)**, besides severe necrosis and cytoplasmic vacuolization of the hepatocytes with inflammatory cell infiltration **(D)**.

The fetal livers treated with 100 mg/kg of CHTiO_2_NPs showed extensive hepatic vacuolation, thrombotic vasculitis of the central vein, and lymphocyte infiltration (Table 5, Figures 4A,B), while those treated with 300 mg/kg of CHTiO_2_NPs showed remarkable congestion and dilation of the blood vessels (Table 5, Figure 4C), as well as severe necrosis and cytoplasmic vacuolization of the hepatocytes with inflammatory cell infiltration (Table 5, Figure 4D).

**Figure 5:**
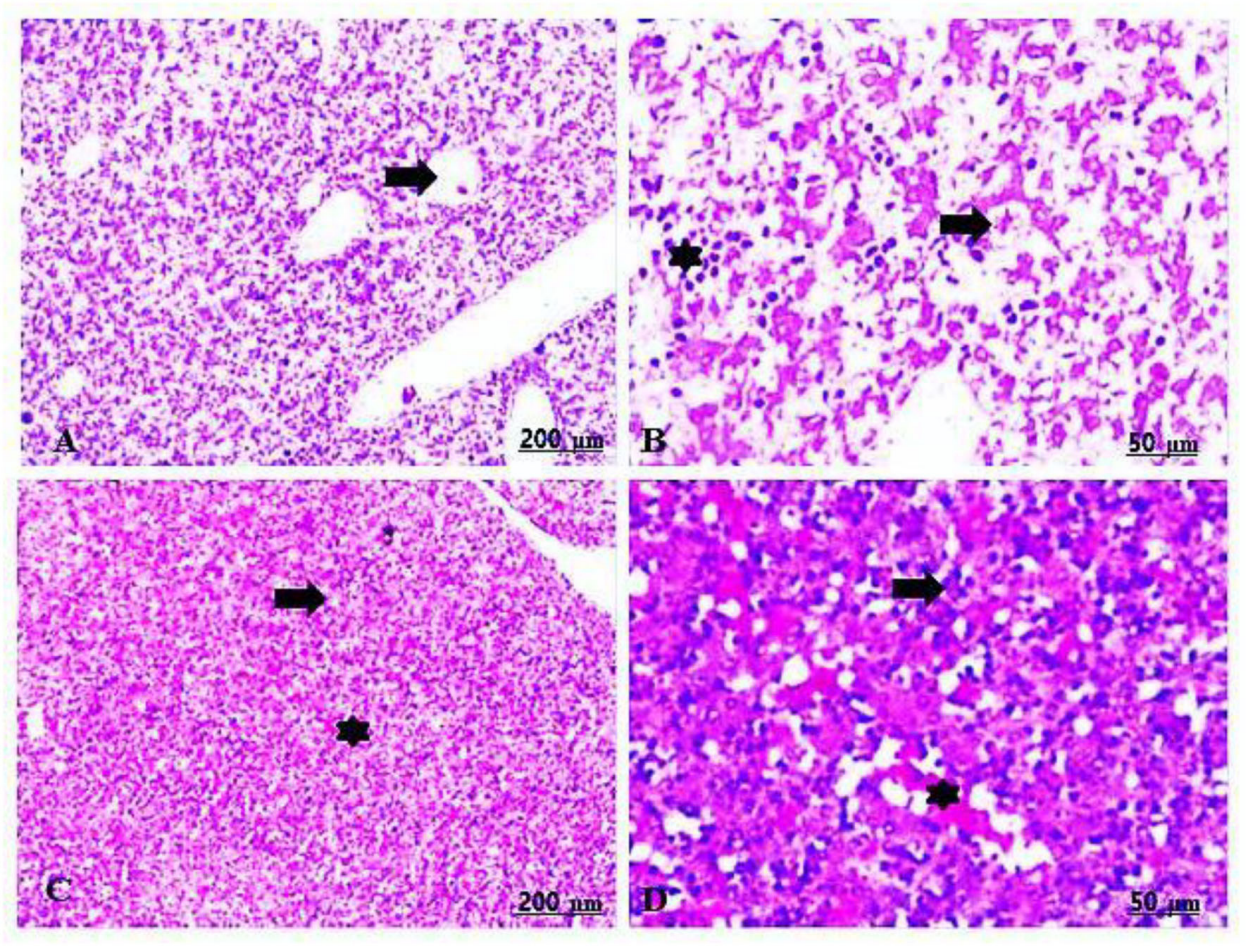
**(A–D)** Light photomicrograph of sections from the fetal liver of GTiO_2_NPs exposed groups. GTiO_2_NPs of 100 mg/kg bwt show slight congestion and dilatation of the central vein **(A)**, in addition to cytoplasmic vacuolation (arrow) and inflammatory cell infiltration (star) **(B)**. GTiO_2_NPs of 300 mg/kg bwt show proliferation of the hepatocytes with mitotic division (arrow) in addition to congestion and dilatation of the blood vessels (star) **(C,D)**.

The fetal livers of the group treated with 100 mg/kg of GTiO_2_NPs exhibited slight congestion and dilatation of the central vein (Table 5, Figure 5A), in addition to cytoplasmic vacuolation and inflammatory cell infiltration (Table 5, Figure 5B). However, the fetal livers that were administered 300 mg/kg of GTiO_2_NPs induced mitotic division of the hepatocytes in addition to congestion and dilatation of the blood vessels (Table 5, Figures 5C,D).

#### Liver of maternal rats

**Figure 6:**
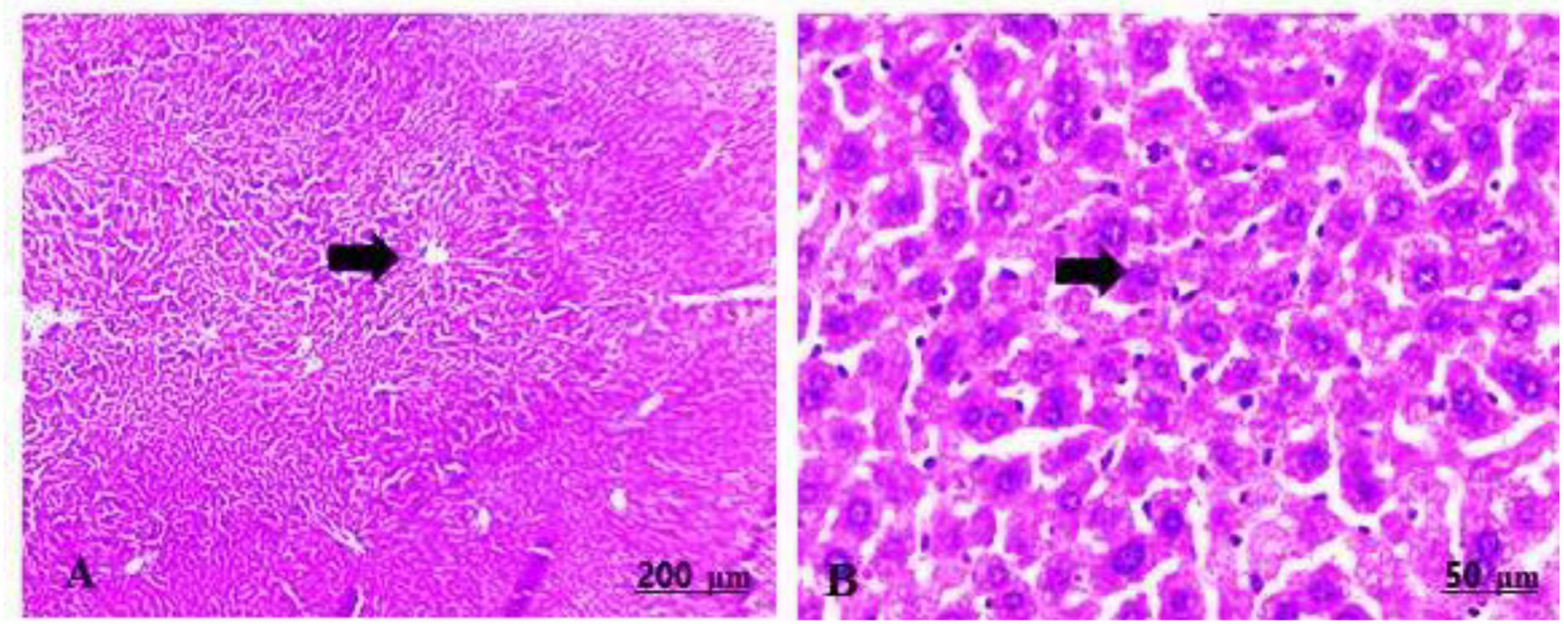
**(A,B)** Light photomicrograph sections from maternal liver of control group showing normally arranged hepatic cords and blood vessels.

The maternal livers of the control group implied a normal arrangement of the hepatocytes and intact vasculature (Table 5, Figures 6A,B).

**Figure 7:**
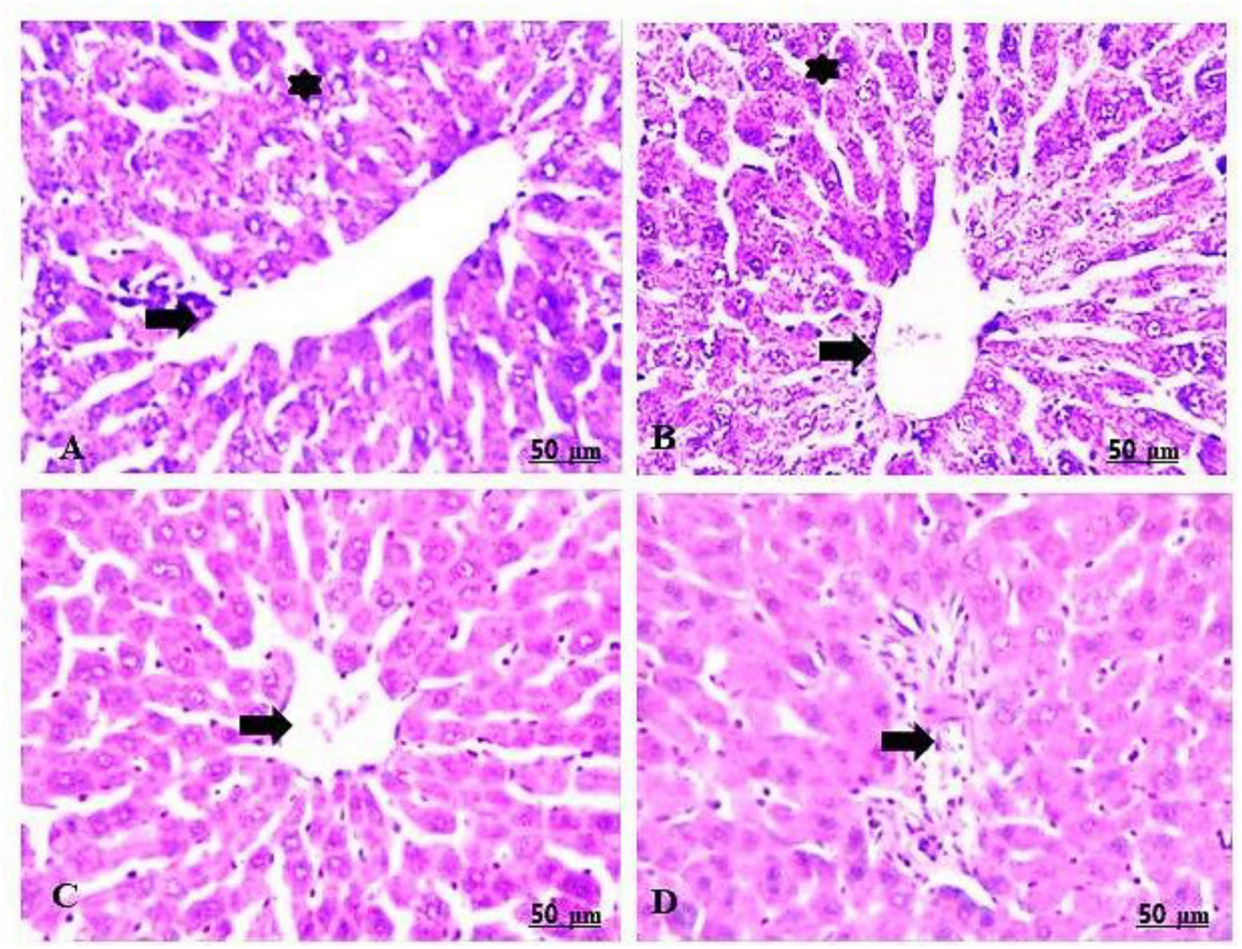
**(A–D)** Light photomicrograph of sections from the liver of CHTiO_2_NPs exposed groups. CHTiO_2_NPs of 100 mg/kg bwt show noticeable dilatation of the central vein with perivascular mononuclear infiltration, mostly lymphocytes (arrow), also prominent vacuolation and necrosis of the hepatocytes (star) **(A,B)**. CHTiO_2_NPs of 300 mg/kg bwt showed congestion of the central vein and blood sinusoids **(C)**, and portal inflammation and fibrosis were seen **(D)**.

The maternal livers of the group treated with 100 mg/kg of CHTiO_2_NPs showed sharp dilatation of the central vein with perivascular cell infiltration, mainly lymphocytes, as well as prominent vacuolation and necrosis of the hepatocytes (Table 5, Figures 7A,B), while the group treated with 300 mg/kg of CHTiO_2_NPs showed congestion of the blood vessels, fundamentally the central vein and blood sinusoids (Table 5, Figure 7C), besides portal fibrosis and inflammation (Table 5, Figure 7D).

**Figure 8:**
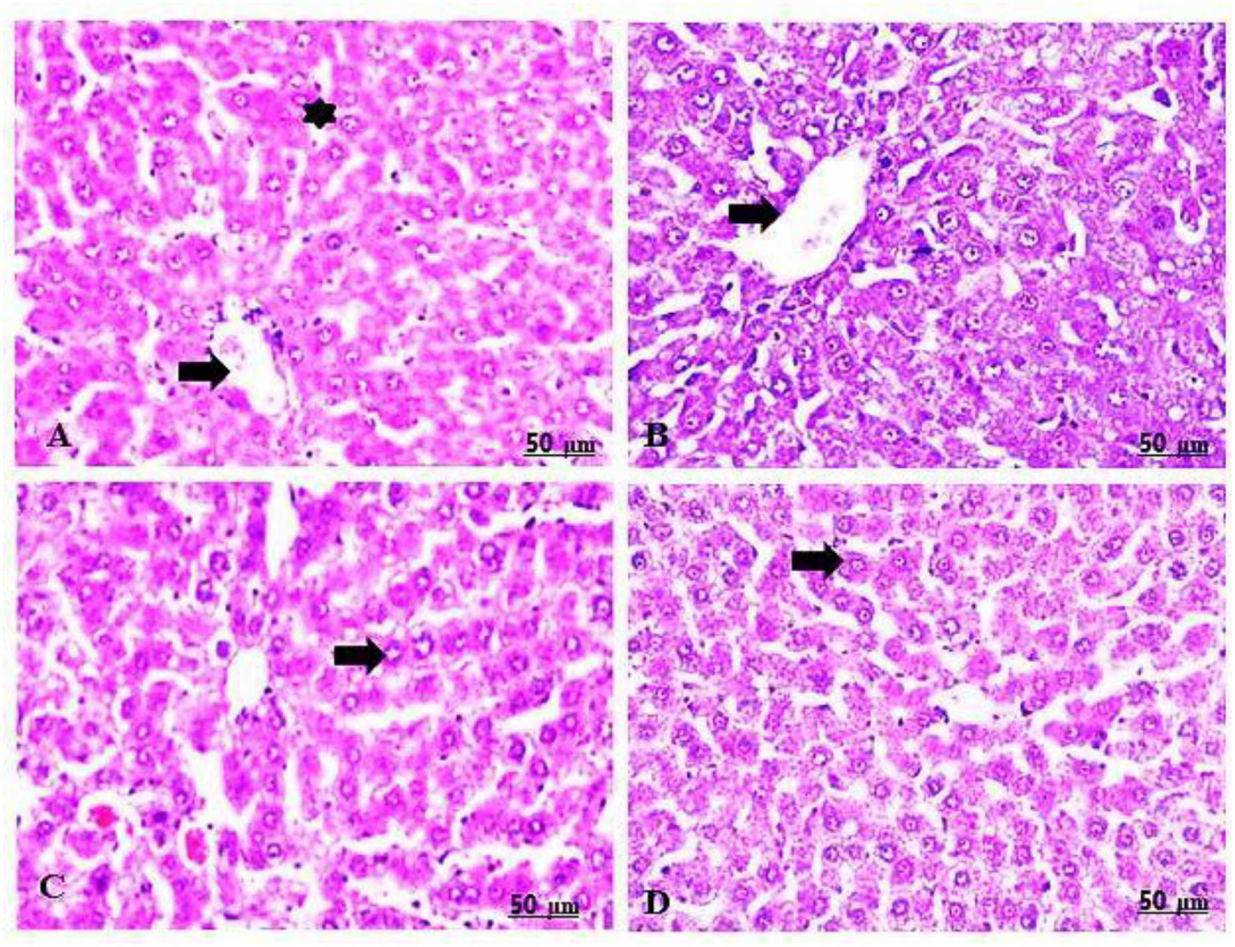
**(A–D)** Light photomicrograph of sections from the maternal liver of GTiO_2_NPs exposed groups. GTiO_2_NPs of 100 mg/kg bwt show slight congestion and dilatation of the central vein (arrow), in addition to the regeneration of some hepatocytes (star) **(A,B)**. GTiO_2_NPs of 300 mg/kg bwt showing regeneration of the hepatocytes in trial to restore normal histological structures **(C,D)**.

The maternal livers in the treated group with 100 mg/kg of GTiO_2_NPs manifested a mild degree of congestion and a dilated central vein, in addition to the regeneration of some hepatocytes (Table 5, Figures 8A,B). Similarly, the maternal livers in the group treated with 300 mg/kg of GTiO_2_NPs elucidated regeneration of the hepatocytes in trials to restore normal histological structures (Table 5, Figures 8C,D).

### Immunohistochemical findings (Bax-IHC stain)

#### Liver of rat fetuses

**Figure 9:**
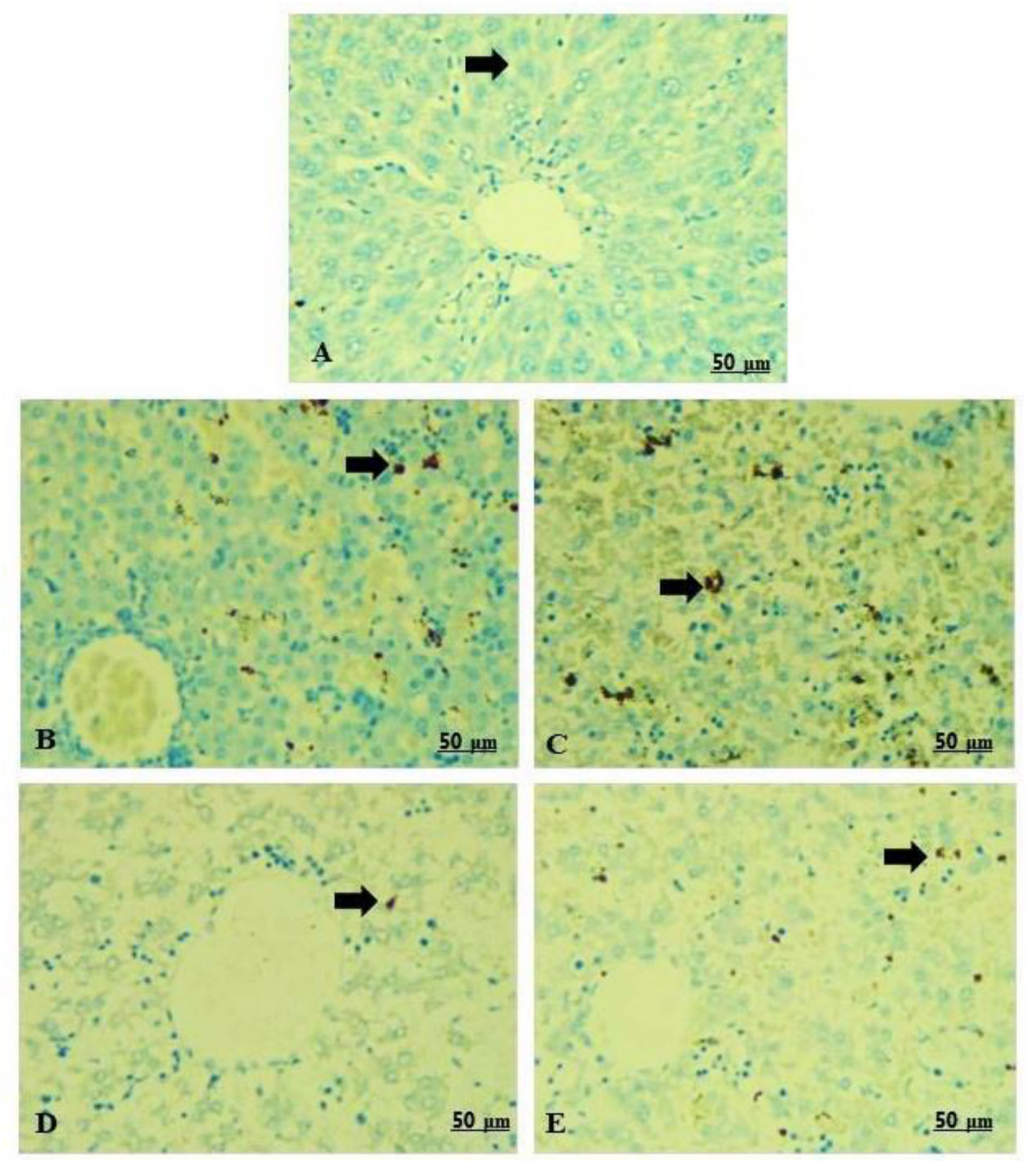
**(A–E)** Light photomicrograph of sections from the fetal liver of control and other exposed groups by Bax-IHC: control showing negative Bax-immunostaining in the hepatocytes **(A)**. Groups 2 and 3 show intense positive staining reactions of Bax in the hepatocytes **(B,C)**. Group 4 shows negative Bax-immunoreactivity in the hepatocytes **(D)**. Group 5 shows a weak positive Bax-immunoreactivity in the hepatocytes **(E)**.

The livers of the rats in the control group showed negative Bax immunoreactivity toward the hepatocytes (Table 5, Figure 9A). In contrast, livers of rats in the 100 and 300 mg/kg CHTiO_2_NPs treated groups expressed a sharp positive Bax immunostaining in the hepatic parenchyma (Table 5, Figures 9B,C). Meanwhile, rats that were administered 100 mg/kg of GTiO_2_NPs by Bax immunostaining showed an apparent negative reaction to Bax in the liver tissues (Table 5, Figure 9D). Moreover, the rats treated with 300 mg/kg of GTiO_2_NPs showed weak Bax immunoassays in the hepatocytes (Table 5, Figure 9E).

## Discussion

Due to its safety, environmental friendliness, simplicity of manufacturing, and low production costs, the green synthesis of nanoparticles has recently attracted much interest (13). The use of antioxidant agents such as plant extracts, natural chemicals, vitamins, and minerals in synthesizing NPs has produced positive results (16). Garlic is well known for its prevalent anti-inflammatory, antioxidant, antibacterial, antiviral, antifungal, and antimutagenic properties (30). Garlic can also offer medicinal and favorable effects such as reducing risk factors for cardiovascular diseases, enhancing the detoxificationprocess, and providing hepatoprotection (31, 32). Its properties have a significant impact on the prevention of many tumors and oral disorders. Garlic pretreatment reduces oxidation, blood glucose, and lipid levels in the liver and the kidney (33). It can actively lower free radical (ROS) levels, enhancing the antioxidant system and defending the body against DNA damage (34).

TiO_2_NPs are one of the most widely used and highly manufactured nanoparticles (35). They are commonly used in our daily lives, for example, in feed additives, the diagnosis of diseases, water treatment, implants, and clothing (36). Unfortunately, most chemically synthesized nanoparticles can cause eco-toxicological problems. As a result, there has been a rise in scientists' interest in the potential effects of nanoparticles on human health and the environment (37). The greenly synthesized nanoparticles have recently been enriched to synthesize several plant extracts (38). The reason for this was to intensify the light on the potential of greenly synthesized TiO_2_NPs using garlic extract to ameliorate chemical TiO_2_NPs induced genotoxicity and hepatotoxicity in female Albino rats.

In this study, garlic extract was successfully used to synthesize TiO_2_NPs. The synthesis of TiO_2_NPs was verified by XRD and HRTEM techniques. The two samples' XRD patterns showed all of the distinctive peaks of the TiO_2_NPs. Furthermore, the lack of diffraction peaks for impurities such as NaCl and Na_2_TiO_3_, which represent the purity and polycrystalline nature of the synthesized samples, was further evidence of the absence of impurities in the two samples' XRD patterns. The reported card supports all recorded peaks from the anatase phase of TiO_2_NPs (JCPDS No. 21-1272), supported by the reported card (39). Garlic extract was used to completely reduce titanium isopropoxide, as evidenced by the production of the anatase phase (40). The higher crystallinity of GTiO_2_ NPs than the CHTiO_2_NPs sample is indicated by the increase in 101-plan intensity. Garlic did not alter the structural characteristics of TiO_2_ because there were no structural variations between the two samples. These results align well with those of sustainable TiO_2_ nanoparticles (17). The crystal size of the produced samples was established by Scherer using the Debye Equation (41):


$$\begin{array}{l} d=\frac{0.89\lambda }{FWHMCos\theta } \end{array}$$

where *d* is the average NPs crystal size, λ is the x-ray source wavelength, a constant crystal shape factor of 0.89, θ is Bragg's diffraction angle, and full width at half maximum (FWHM) of angular XRD peaks was recorded at diffraction angle 2θ. The difference between the estimated crystal sizes of CHTiO_2_NPs and GTiO_2_NPs, which was increased to 53.31 nm from 48.11 nm, may be due to the electrostatic attraction between the biomolecules on the NP's surface (42). Due to the biomolecules on its surface, there are no harmful effects from exposure to GTiO_2_NPs. According to reports, the bioactivity of NPs produced through green syntheses, such as their ability to combat cancer and germs, is greatly enhanced by the capping agents provided by biomolecules (36, 43, 44).

Nanoparticles can be toxic to cells and tissues both *in vivo* and *in vitro*, depending on their dose and size (45). TiO_2_NPs are easily ingested by humans through a variety of methods and can disrupt the metabolism. The hepatotoxicity of TiO_2_NPs was deeply investigated by many researchers (11, 46–48). The main mechanism is the production of cellular oxidative stress, which in turn oxidizes the unsaturated lipids in the cell membrane, causing cellular leakage and liver's functional cell integrity loss (49). Compared to male rats, female rats were more susceptible to the toxicity of TiO_2_NPs. Recent research indicated that oxidative stress may be intimately related to this damage. The different antioxidant capacities of male and female rats may contribute to gender disparities.

Additionally, TiO_2_NPs taken orally could have several harmful health effects, such as liver damage, without appearing in the blood or other internal organs (50). This can be observed by the significant increase in the liver enzyme levels of ALT, AST, ALP, total bilirubin, and protein in groups 2 and 3. On the contrary, groups 4 and 5, treated with green TiO_2_NPs, showed a significant decrease in the AST level, which can be attributed to the antioxidant activity of garlic extract (34).

Garlic includes water-soluble organ sulfur compounds (OSCs) such as cycloalliin and S-allyl cysteine and oil-soluble OSCs like diallyl sulfide. According to a recent report, garlic-derived OSCs are responsible for the hepatoprotective effects of garlic (51). These OSCs have been recorded to enhance antioxidants such as glutathione, catalase, and GSH peroxidase (52). According to one study, pretreatment with aqueous garlic extract helped to mitigate the liver damage and oxidative stress caused by galactosamine/lipoploysaccharide (53).

The garlic extract has a sufficient proportion of polyphenols, sterols, and tocopherols. These active ingredients were employed in the hepatoprotective inquiry study and have been shown to have antioxidant, hypoglycemic, and cholesterol-lowering properties (54).

The histological studies confirmed the biochemical results. Hepatic abnormalities were noticed in the livers of maternal and embryo-fetal rats exposed to CHTiO_2_NPs in a dose-dependent manner. The observed hepatic abnormalities were congestion, dilatation of the blood vessels, necrosis, vacuolization, and inflammatory cell infiltration. The discovery of inflammatory cells in the liver suggests that TiO_2_NPs interact with enzymes and other proteins in the interstitium, disrupt the antioxidant defense system, and produce excessive amounts of ROS, leading to inflammatory conditions and blood vessel dilation because TiO_2_NPs disturb the permeability of cell membranes in hepatocytes and the endothelial lining of blood vessels (55). However, these abnormalities were reduced in the groups treated with GTiO_2_NPs linked with hepatocyte generation enhancement in a dose-dependent manner. These ameliorative effects could be attributed to garlic's bioactive compounds, such as diallyl sulfide, allicin, ajoene, and allium. These compounds are directly related to oxidative stress induction, lipid peroxidation, inflammatory responses, and apoptotic effects (31, 32).

Several conserved domains known as Bcl-_2_ homology (BH) are found in the Bcl-_2_ family of proteins. High homology is found in the BHs domain of the pro-apoptotic Bcl-2 family members Bax and Bak (56). The overexpression of the Bax protein causes apoptotic cell death (57). The immunohistochemical results reflect a positive detection of Bax in groups treated with both 100 mg/kg of CHTiO_2_NPs and 300 mg/kg of CHTiO_2_NPs, compared to a negative detection in the 100 mg/kg of GTiO_2_NPs, 300 mg/kg of GTiO_2_NPs, and the control group. These results suggested that the internalization of CHTiO_2_NPs produces ROS that disrupts the cellular redox balance, speeds up cell death and lipid peroxidation, changes gene expression, attaches to the mitochondrial membrane, and causes DNA damage and apoptosis in cells (58–61).

## Conclusion

Garlic extract was successfully used to synthesize TiO_2_NPs. The produced TiO_2_NPs showed a sort of agglomeration with a semispherical shape and no toxic effect on HepG_2_ under our experimental conditions. Maternal exposure to chemically synthesized TiO_2_ NPs at higher doses induced a significant decrease in the maternal and fetal body weights, contrary with a significant increase in the mean level of serum AST and ALT activity and total protein level, with a remarkable histological alteration in the fetal and maternal livers. In addition, distinct positive staining of Bax is expressed in the hepatocytes, which directly indicates liver damage in both maternal and embryonic rats. Nevertheless, biosynthesis of TiO_2_NPs using garlic extract could mitigate the altered parameters and minimally affect the liver's normal architecture. Depending on the discussed findings, it could be concluded that the bioactivity of TiO_2_NPs can be modified with green synthesis using garlic extract. Compared to CHTiO_2_NPs, the exposure to GTiO_2_NPs showed reduced damage in the liver tissues of maternal and embryo-fetal rats.

## Data availability statement

The original contributions presented in the study are included in the article/supplementary material, further inquiries can be directed to the corresponding authors.

## Ethics statement

Animal Experimental Guidelines were followed, and the Animal Care and Use Committee of the Animal Health Research Institute, Faculty of Science, South Valley University, Qena, Egypt, approved the experimental procedures (approval no. 002/9/22).

## Author contributions

ZK and AAE jointly developed the hypothesis and concept of the study, contributed to the chemical and material preparation, and the techniques performed. For this research and scientific paper, ZA-A, AHS, FZ, SO, AAM, and IFR were involved in the experimental procedures and analyses. All authors revised, edited, read, and approved the final manuscript.
